# Increase of ADAM10 Level in Coronary Artery In-Stent Restenosis Segments in Diabetic Minipigs: High ADAM10 Expression Promoting Growth and Migration in Human Vascular Smooth Muscle Cells via Notch 1 and 3

**DOI:** 10.1371/journal.pone.0083853

**Published:** 2013-12-27

**Authors:** Ke Yang, Lin Lu, Yan Liu, Qi Zhang, Li Jin Pu, Lin Jie Wang, Zhen Bing Zhu, Ya. Nan Wang, Hua Meng, Xiao Jie Zhang, Run Du, Qiu Jing Chen, Wei Feng Shen

**Affiliations:** 1 Institute of Cardiovascular Diseases, Medical School of Jiaotong University, Shanghai, People’s Republic of China; 2 Department of Cardiology, Rui Jin Hospital, Medical School of Jiaotong University, Shanghai, People’s Republic of China; Facultad de Medicina, Universidad Autonoma Madrid, Spain

## Abstract

**Background:**

This study aimed to identify major proteins in the pathogenesis of coronary artery in-stent restenosis (ISR) in diabetic minipigs with sirolimus-eluting stenting, and to investigate the roles of key candidate molecules, particularly ADAM10, in human arterial smooth muscle cells (HASMCs).

**Methods and Results:**

The stents were implanted in the coronary arteries of 15 diabetic and 26 non-diabetic minipigs, and angiography was repeated at six months. The intima of one vascular segment with significant ISR and one with non-ISR in diabetic minipigs were isolated and cultured in conditioned medium (CM). The CM was analyzed by LC-MS/MS to uncover proteins whose levels were significantly increased (≥1.5-fold) in ISR than in non-ISR tissues. After literature searching, we focused on the identified proteins, whose biological functions were most potentially related to ISR pathophysiology. Among them, ADAM10 was significantly increased in diabetic and non-diabetic ISR tissues as compared with non-ISR controls. In cell experiments, retrovirus-mediated overexpression of ADAM10 promoted growth and migration of HASMCs. The effects of ADAM10 were more remarkable in high-glucose culture than in low-glucose culture. Using shRNA and an inhibitor of γ-secretase (GSI), we found that the influences of ADAM10 were in part mediated by Notch1 and notch 3 pathway, which up-regulated Notch downstream genes and enhanced nuclear translocation of the small intracellular component of Notch1 and Notch3.

**Conclusions:**

This study has identified significantly increased expression of ADAM10 in the ISR versus non-ISR segment in diabetic minipigs and implicates ADAM10 in the enhanced neointimal formation observed in diabetes after vascular injury.

## Introduction

It is clear that sirolimus- and paclitaxel-eluting stents are less effective in the prevention of in-stent restenosis (ISR) in patients with diabetes as compared to those without diabetes [1]. To investigate the mechanisms of ISR in diabetes, we implanted sirolimus-eluting stents into the major coronary arteries in minipigs with streptozotocin-induced diabetes (diabetic group, stent number =30) and in non-diabetic controls (non-diabetic group, stent number = 52) [2]. After 6 months, a significant higher ISR ratio was observed by angiography in diabetic (20%) than in non-diabetic group (7.7%). 

One vascular segment with significant ISR (diameter stenosis ≈ 70%), and one with non-significant ISR (diameter stenosis ≈ 20%) from diabetic group were employed for proteomic analysis (Experiment flowchart shown in Figure **S1**). The intima was isolated and cultured in serum-free DMEM medium. The conditioned medium (CM) from intima sections was analyzed by LC-MS/MS and label-free quantification to compare proteins between ISR and non-ISR intima. Among identified proteins whose levels were significantly changed in ISR tissues (≥1.5 fold increase or ≥1.5 fold decrease) than in non-ISR tissues, and also based upon information regarding biological functions from literatures, we noted several proteins that are most potentially related to the pathophysiology of ISR ( Table **S1** and **S2**). Among these proteins of increased levels, adipocyte fatty acid binding protein (AFABP) has been uncovered to be significantly increased in ISR versus non-ISR segments, and to promote proliferation and migration in human aortic smooth muscle cells (HASMCs) by our group [3]. The other proteins have been suggested to promote inflammation, ROS production, cell proliferation, cardiovascular remodeling, neurodegeneration and tumor growth, such as A disintegrin and metalloproteinase (ADAM)10, toll-like receptor 4, cyclophilin A and S100A11, etc.

ADAM10, an α-secretase in the processing of the amyloid precursor protein [4], has multiple substrates, including Notch [5-10]. The Notch receptor pathway functions to maintain vascular homeostasis and angiogenesis. Engagement of Notch receptor with ligands leads to shedding of Notch ectodomain by ADAM10. After cleavage, γ-secretase further releases a small intracellular part (IC) of Notch, which then translocates to the nucleus and acts as a transcription factor [11-13]. Of the 4 Notch receptors, Notch1 and Notch3 are predominant in vascular smooth muscle cells (SMCs). We found ADAM10 levels to be significantly increased in ISR than in non-ISR intima from both diabetic and non-diabetic groups. Since SMCs are the principal cell type within the expanding neointima, and as a recent study demonstrated that Notch1 mediates SMC proliferation and neointimal formation after vascular injury [14], in this work, we sought to test the impact of ADAM10 in HASMC properties. To the best of our knowledge, this study is the first to show the increase of ADAM10 in coronary artery ISR tissue of porcine model, with the influence of ADAM10 overexpression clarified.

## Materials and Methods

### Materials

The in solution γ-Secretase Inhibitor IX was purchased from MERCK (Darmstadt, Germany) and ADAM10 antibody was from Abcam (Cambridge, UK). Antibodies for detection of Notch1, Notch1 IC, Notch2 IC, Notch3 IC, and the secondary antibody were from Cell Signaling Technology (Danvers, Massachusetts, USA). Activated Notch4 antibody was from Millipore (Billerica, MA, USA). Streptozotocin and 3-(4, 5-dimethylthiazol-2-yl)-2, 5-diphenyltetrazolium bromide (MTT), as well as other chemicals and reagents were obtained from Sigma-Aldrich (St Louis, MO, USA). The BrdU cell proliferation assay kit was obtained from Calbiochem (Merck, Darmstadt, Germany). Insulin Novolin^@^ 30R was purchased from Novo Nordisk A/S (Bagsværd, Denmark). Aspirin was from Bayer (Morristown, NJ, USA), and ticlopidine was from Sanofi-Aventis (Paris, France). AGE-BSA was from Calbiochem (Merck, Darmstadt, Germany).

### Animal models, anesthetic protocol, stenting procedure and angiographic examination

All experiments were conducted according to European Union Directive number 86/609/CEE for the use of animals in research, and the approval was granted by ethics review board of Shanghai Jiaotong University School of Medicine. The detailed procedure was reported previously [2]. Briefly, 41 minipigs (male, body weight, 20-25 kg) were raised in separate pens. Diabetes mellitus was induced by intravenous administration of streptozotocin (125 mg/kg) in 15 minipigs [15,16]. Insulin therapy was given to maintain a fasting glucose level below 10 mM. Another 26 minipigs were placed in the non-diabetic group. Significant difference was documented in fasting plasma glucose levels between diabetic and non-diabetic group at 1 and 6 months (8.0±3.06 vs. 2.3±1.02 mM, and 7.09±4.01 vs. 2.1±1.1 mM, respectively, both P<0.001). 

The entire experimental procedure is outlined in Figure **S1**. Coronary angiography and stent implantation were performed two weeks after induction of diabetes. Aspirin (300 mg/day) and Ticlopidine (250 mg/day) were given 2 days before catheterization, and continued to the end of the experiment. After an overnight fast, the animals were sedated by ketamine hydrochloride (20 mg/kg) and midazolam (1 mg/kg) intramuscularly. Anesthesia was induced with sodium thiopental (12.5mg/kg) intravenously and isoflurane (1mg/kg) by mask. After tracheal intubation, the tube was connected to the anesthesia equipment (Hewlett-Packard, Palo Alto, California). Anesthesia was maintained with mechanical ventilation of oxygen (100%) and isoflurane (1.5% to 2.5%) and morphine sulphate at constant-rate infusion of 0.2 mg/kg intravenously, with monitoring of heart rate and blood pressure. The minipigs were ventilated at a rate of 10 to 12 breaths per minute and tidal volume was adjusted to maintain the end-tidal carbon dioxide concentration in the expired air at 33 mmHg. 

Through the right femoral artery, a 6F Amplatz right coronary guiding catheter was introduced to engage both right and left coronary arteries. For each animal, 2 sirolimus-eluting stents (Cypher), 18 mm in length and 2.5 mm or 3.0 mm in diameter, were implanted. After the surgical procedure, the animals were remained intubated until spontaneous respiration recovered. Aspirin and ticlopidine were given to the end of study in all animals. 

Since peak restenosis of bare metal stent-treated segment in patients is observed at 3 months and remains stable at 6 months, and drug eluting stent delays completion of vessel reparative process [17,18], our experience also suggested that 6 months time was required for ISR formation in minipig models, repeat angiography was therefore performed after 6 months, and ISR was defined as 50% luminal diameter narrowing. Meticulous care was taken to obtain coronary angiography at similar projections, as possible. Intravascular ultrasound (IVUS) imaging was made to evaluate the degree of neointimal hyperplasia in all stented vessels. All IVUS images were acquired with automated pullback at 0.5mm/s after intracoronary nitrates using a commercially available imaging system (Galaxy II, Boston Scientific).

After completion of the experiments, the minipigs were euthanized. The animals were premedicated with midazolam hydrochloride intravenously, and then anesthetized and euthanized by intravenous overdose of sodium thiopental and potassium chloride.

Offline quantitative analyses of procedural, post-procedural, and follow-up angiography were performed by an independent core laboratory (Terra, GE, Niskayuna, NY). All imaging analysis included the stented segment as well as their margins, 5mm proximal and distal to the stent. Late loss was defined as the difference between the minimal lumen diameter immediately after procedure and at 6 months. 

### Explant tissue culture and identification of proteins by LC-MS/MS

In order to identify the proteins in the intima, and also to bypass the masking effects of structural proteins, we employed the proteomic strategy to analyze the tissue CM. Briefly, on the basis of the quantitative angiographic results, one segment sample with ISR (diameter stenosis ≈ 70%) and one with non-ISR (diameter stenosis ≈ 20%) were chosen to perform the discovery experiment. The intima of these segments were isolated and washed with saline before they were finely minced into 2-5 mg pieces. The tissue pieces were incubated in a Petri dish containing serum-free DMEM, supplemented with 50 µg/ml gentamycin for 24h. 

Then, the CM was collected, and protein concentration was measured using Bradford assay (Bio-Rad, Hercules CA). The proteins from CM were further fractionated by SDS-PAGE on a 9 % gel, and visualized by Coomassie Brilliant Blue G-250 based staining. The whole lane was excised and cut into 4 pieces to separate high-abundance serum proteins and low-abundance proteins in CM. Each piece was washed in pure water and dehydrated in acetonitrile to remove residual SDS and Coomassie Blue. In-gel reduction was performed with dithiothreitol (10 mM) for 1 h at 60 °C, and carbamidomethylation with iodoacetamide (55 mM) for 45 min at room temperature in the dark. Trypsin (0.1 µg/50 mM ammonium bicarbonate) was added and protein digestion was performed overnight at 37 °C. 

LC-MS/MS analysis was carried out using the Proteome X workstation coupled with LTQ linear ion trap mass spectrometer (Thermo Electron, San Jose, CA) equipped with a RP-C18 microcapillary column (Column Technology Inc, Fremont CA). 

The mobile phases were 0.1% formic acid (A) and 84% acetonitrile in 0.1% formic acid (B). The flow rate was maintained at 200 nL/min. The gradient was started at 4% B, reached 50% in 110 min, 50-100% B in the next 5 min, and then 100%B in the final 6 min. MS/MS spectra were acquired from the most three intense ions from the full MS, and MS peaks were quantified using “Two-Dimensional Image Converted Analysis of Liquid chromatography and mass spectrometry (2DICAL)” [19]. The MS data obtained were interpreted using SEQUEST against the UniProtKB swine protein sequence database for porcine protein identification. DeCyder^TM^ MS Differential Analysis Software (DeCyder MS, GE Healthcare) was used to visualize and analyze the signal intensity maps from the LC-MS/MS experiments. 

### Retrovirus construction and transfection

Human ADAM10 cDNA was amplified by RT-PCR from human umbilical endothelium cell mRNA, and ligated to TA cloning vector (Invitrogen, Carlsbad, California). After confirmation by sequencing, the amplified ADAM10 cDNA was transferred to the pLXSN.retro vector (Clontech, Mountain View, CA). To construct shRNA of human ADAM10, Notch1, and Notch3, synthesized sense and antisense sequences for shRNA were annealed and inserted into the pSIREN.Retro.Q vector (Clontech, Mountain View, CA), according to the manufacturer’s instructions. The primers flanking the coding region of ADAM10 and targeted sequences for ADAM10, Notch1, and Notch3 knockdown were listed in Table **S3**. To generate retroviral supernatant, the recombinant plasmids were transfected into PT67 packaging cells (Clontech, Mountain View, CA) using lipofectamine-2000 (Invitrogen). The cell culture supernatants containing the retrovirus were harvested and immediately used for infection or stored in -80°C until use.

### Cell culture and retrovirus infection

Human aortic smooth muscle cells (HASMC) were obtained from Cascade Biologics Inc. (Portland, OR, United States). The cells were grown in M199 medium (low-glucose or high-glucose), supplemented with 50 µg/mL gentamycin, 50 µg/mL amphotericin-B, and 10% FBS (Gibco BRL, Life Technologies, Inc). Retrovirus was added to the cells with a MOI (multiplicity of infection) of 20 plaque-forming units per cell in complete medium plus polybrene (8 µg/ml) for 24 hours. HASMCs were infected with retroviral stocks and selected with G418 (Sigma, St. Louis, MO) or puromycin (Sigma, St. Louis, MO). Stable cell lines were established after 1-week selection. Transient expression of siRNA to knockdown Notch1 and Notch3 in HASMCs was achieved using Lipofectamine 2000 (Invitrogen, Carlsbad, California) according to the protocol. Cells were harvested after 48h and the expression of relevant proteins was examined by Western blot analysis.

### MTT assay

Cell proliferation was examined by MTT assay. Briefly, cells (5×10^3^/well) were plated in 96-well plates, and then the medium was changed to fresh medium with or without addition of factors. At 24h, 20 μL of 5 mg/ml MTT solution was added to each well and the cells were incubated for an additional 4 hours. Thereafter, 150 μL of DMSO were added to each well, and an absorbance was read at 490 nm on a Microplate reader, Model 680 (Bio-Rad, Hercules, CA).

### BrdU cell proliferation assay

Cell proliferation was also measured with 5-bromo-2’-deoxy-uridine (BrdU) labeling and a Detection kit II (Roche Diagnostics Co., Indianapolis, IN), following the manufacturer’s instructions. Briefly, cells (5×10^3^/well) were plated on 96-well plates, with or without addition of factors. After 24h, 10 µM Brdu was added to the medium for additional 4-hours of culture. The cells were fixed and incubated in anti-BrdU antibody for 1h, HRP-conjugated secondary antibody for 30 min, and underwent reaction with TMB substrate for 15 min. Within 30 minutes after adding ELISA stop solution, the OD values were measured at 450-540 nm by a Microplate Reader, Model 680 (Bio-Rad, Hercules, CA).

### Wound-healing assay

Cells were grown to confluence in 6-well plates and starved for 24 h. A scratch was made with a sterile tip and the cells were then incubated in fresh Medium 199 with or without addition of factors. The starting point was marked with a marker pen at the bottom of the plate. At 0 and 24 h, images were taken (Olympus, Shinjuku, Tokyo, Japan). The wound width of each dish was measured using Image-Pro Plus Version 6.2 (Media Cybernetics, MD, USA). 

### Cell migration assay

Cell migration was assessed by Boyden chamber assay including a 48-well transwell plate (Millipore, MA, USA). Cells at 70-80% confluence were starved overnight in serum-free medium, trypsinized, and resuspended in serum-free medium (3×10^5^ cells/300 µl). The cell suspension was added to the upper chamber, and the bottom chamber was filled with Medium 199 containing 10% FBS medium. The chamber was incubated at 37°C in a CO_2_ incubator for 6-8 h, then the filter was removed and non-migrated cells were scraped from the upper surface. The migrated cells were stained and treated with extract buffer, and the number of cells was then quantified by OD 560 nm measurement. 

### Quantitative real-time RT-PCR

Total RNA was extracted using a Trizol reagent (Invitrogen, Carlsbad, CA). Two microgram of total RNA per sample was reverse-transcribed by the AMV Reverse Transcription System (Promega, Madison, WI). Real time PCR amplification was performed with SYBR Green PCR Master Mix (Santa Cruz, CA) in a StepOne PCR amplifier (Applied BioSystems, Foster City, CA). The relative quantitative value was expressed by the ΔΔCT method. Uninfected cells were used as the reference sample and β-actin was used as the endogenous control. Each experiment was performed in duplicates and repeated six times. The primer sequences and expected products size are listed in Table **S4**. Gene expression levels were normalized with beta-actin, and data was analyzed with StepOne software v2.1 (Applied BioSystems, Foster City, CA).

### Western blot

Cells were harvested with the RIPA solution (Fermentas, MD, USA). Equal amounts of protein extracts were subjected to 6^~^12% SDS/PAGE and transferred to a polyvinylidene difluoride membrane. The membrane was blocked and probed overnight at 4°C with primary antibodies, followed by incubation with HRP peroxidase-conjugated secondary antibodies for 1 h at room temperature. After washing, the membrane was processed using Immobilon^TM^ Western chemiluminescent HRP substrate (Millipore, Billerica, MA). Beta-actin was employed as an internal control. Each image was captured and the density of each band was analyzed with GelDoc software (Bio-Rad, Munich, Germany). 

### Immunofluorescence

Cells were grown on coverslips and fixed with 4% paraformaldehyde for 10 minutes at room temperature, and then treated with 0.1% Triton-X100 for 10 minutes. After three 5-minutes washes in PBS, cells were blocked by 5% donkey serum for 1h and probed overnight at 4°C with primary antibodies; this was followed by incubation with CY3 fluorescence conjugated secondary antibody for 30 minutes at room temperature and a subsequent staining of DAPI for 10 minutes. Cells were mounted in moiwol, an anti-fade agent, and visualized with a fluorescence microscope (DP72, Olympus, Shinjuku, Tokyo, Japan). 

### Statistical Analysis

Data are presented as mean ± SD or SEM. In vitro cell experiments were repeated a minimum of six times. Differences between groups were tested using one-way ANOVA, with post-hoc Dunnet C test. Two-sided probability level of P <0.05 was considered statistically significant. All analyses were done with SPSS for Windows 13.0.

## Results

### Significant neointimal hyperplasia observed in STZ-induced diabetic minipigs as compared with non-diabetic minipigs after six months of intracoronary stenting

15 diabetic minipigs and 26 non-diabetic minipigs were included in this study. Two animals in the diabetic group and one in the non-diabetic group experienced ventricular fibrillation during stenting and were rescued by electrical shock. In follow-up, 2 diabetic minipigs had pulmonary infection and recovered with antibiotic treatment. All animals survived until the end of the study. 

 The distribution of stents and angiographic and IVUS features at 6 months are listed in Figure **S1** & Table **S5**. The ISR rate was higher in the diabetic group than in the non-diabetic group, but did not reach statistical significance (20% vs. 7.7%, P>0.05). However, remarkable differences were observed in minimal lumen diameter, late loss and neointimal volume between diabetic and non-diabetic animals in follow-up examinations (all P<0.05). 

### Proteomic analysis of the conditioned medium of ISR and non-ISR tissue in diabetic minipigs uncovering proteins with significantly altered levels

Intima from one ISR segment and one non-ISR segment of diabetic minipigs was used for explant tissue culture. Protein expression profiling from the two CM exhibited homogeneity from proteomic studies (data not shown). After LC-MS/MS and 2DICAL analysis, we identified 659 porcine proteins, among which 163 proteins had significantly higher levels (≥1.5-fold increase) and 127 proteins displayed remarkably lower levels (≥1.5-fold decrease) in ISR tissue than in non-ISR tissue. After literature search regarding to the biological functions, we noted several significantly enhanced proteins with biological significances being very potentially related to ISR pathophsiology. These proteins included ADAM10, AFABP, Heart-type FABP, aldose reductase, apolipoprotein B, bone morphogenetic protein 1, cyclophilin A, high mobility group protein B1, macrophage migration inhibitory factor, S100A11, Thrombospondin-1, TNF receptor-associated factor 6, toll-like receptor 4, vinculin, vitronectin, and 14-3-3 protein gamma, with major biological effects listed in Table **S1**. Moreover, several proteins exhibiting low levels were inferred to be likely related to ISR, such as apolipoprotein A-I, gelsolin, paraoxonase 1 and selenium-binding protein 1, etc (Table **S2**). 

### Increase of ADAM10 levels and activities detected in ISR than in non-ISR segments in diabetic minipigs

**Figure 1:**
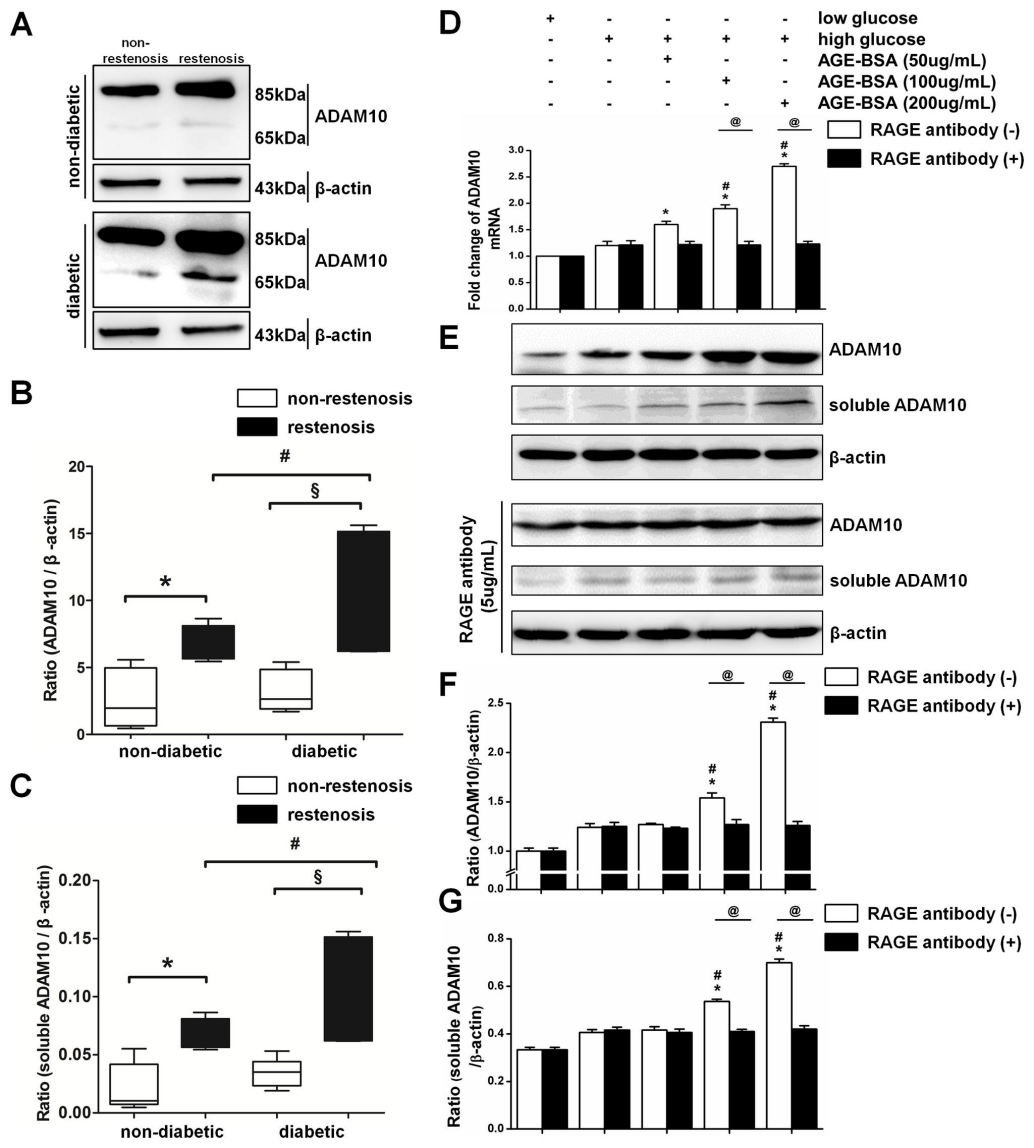
ADAM10 is significantly increased in ISR vs. **non-ISR intima in minipigs**. A, Western blot assay showed ADAM10 and soluble ADAM10 levels in intima of non-diabetic (ISR samples, n=4; non-ISR samples, n=48; for each sample, 3 replicates were performed) and diabetic minipigs (ISR samples, n=6; non-ISR samples, n=24; for each sample, 3 replicates were performed). β-actin was used as internal control. **B** and **C**, quantification of ADAM10 and soluble ADAM10 levels in **A**. *P<0.05 vs. non-diabetic non-ISR; §P<0.05 vs. diabetic non-ISR; # P<0.05 vs. non-diabetic ISR. **D**, HASMCs were treated with low glucose (5 mM), high glucose (25 mM), and high glucose with addition of increasing AGE-BSA (50, 100, and 200 ug/ml) in the presence or absence of anti-RAGE antibody (5 ug/ml). The mRNA level of ADAM10 was examined by real-time PCR in these hASMCs. *P<0.05 vs. low glucose; #P<0.05 vs. control cells cultured in high glucose; @P<0.05. **E**, western blot was performed to examine ADAM10 and soluble ADAM10 levels in the cells in **A**, with β-actin being used as internal control. **F** and **G**, quantification of ADAM10 and soluble ADAM10 levels respectively in E. The symbols of comparison were same as in D.

To verify the results of proteomic analysis on ADAM10, we examined its expression in the intima of stented segments in diabetic minipigs. ADAM10 and soluble ADAM10 protein levels were significantly increased in ISR (n=6) than in non-ISR segments (n=24) (P<0.05), as shown in Figure **1A, B, C**. We also observed significantly higher ADAM10 and soluble ADAM10 levels between ISR (n=4) and non-ISR segments (n=48) in the non-diabetic minipigs. Notably, compared to non-diabetic ISR segments, diabetic ISR segments exhibited significantly higher levels of ADAM10 and soluble ADAM10 protein (P<0.05) (Figure **1A, B, C**). The activation of ADAM10 has been detected by commercially available kit (ANASPEC Inc, CA, USA), we detected consistent changes of ADAM10 activities in ISR versus non-ISR tissue (Figure **S2**)

Based on these findings, we sought to explore if ADAM10 might be regulated by high glucose conditions, which are pathognomonic of the diabetic state. Hence, we treated HASMCs with high glucose (25 mM D-glucose) vs. low glucose (5 mM D-glucose) conditions. Incubation of HASMCs with high glucose induced an elevation in mRNA and protein levels of ADAM10 as compared to low glucose (for all comparison, P<0.05). This effect was further enhanced dose-dependently by the combination of high glucose and advanced glycation endproducts (AGEs) versus high glucose alone (P<0.05) (Figure **1D, E, F, G**). However, the presence of anti-RAGE antibody significantly attenuated AGEs-induced enhancement of ADAM10 (P<0.05). 

### Promotion of ADAM10 overexpression on the growth and migration of HASMCs

**Figure 2:**
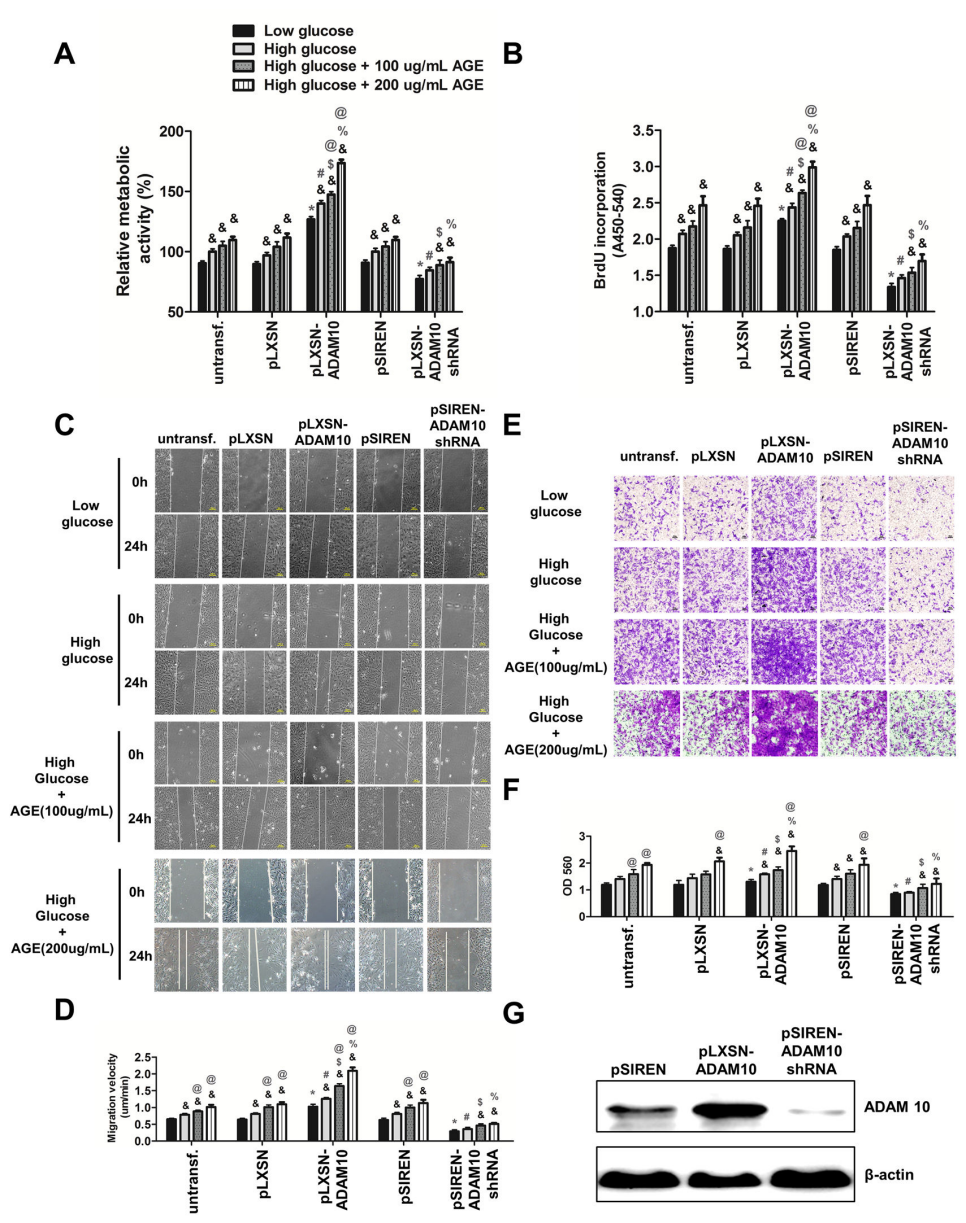
Overexpression of ADAM10 promotes proliferation and migration of HASMCs in low glucose, high glucose and high glucose medium with addition of AGE-BSA (100 and 200 µg/ml). **A**, vector-transduced, ADAM10-overexpressing and ADAM10-silenced HASMCs were generated by retrovirus-mediated gene transfer and selection. These HASMCs were grown in low glucose, high glucose and high glucose with addition of AGE-BSA (100 and 200 ug/ml). MTT assay was performed to test the cell viability. *P<0.05, vs. untransfected cells in low glucose medium; #P<0.05 vs. untransfected cells in high glucose medium; $P<0.05 vs. untransfected cells in high glucose medium with addition of AGE-BSA (100ug/mL); %P<0.05 vs. untransfected cells in high glucose medium with addition of AGE-BSA (200ug/mL); & p<0.05, vs. the same kind of cells cultured in low glucose; @P<0.05, vs. the same kind of cells in high glucose. **B**, BrdU proliferation assay was done to test the cell proliferation ability. The symbols of comparison were same as in A. C, wound healing assay was performed to analyze the migration velocity (µm/min) of HASMCs in low glucose, high glucose and high glucose medium with AGE-BSA. The images were taken before and 24 h after scratch. **D**, quantification of the data in **C**, the symbols of comparison were same as in A. **E**, transwell assay was used to test the migration ability of HASMCs. The cells were added to the upper chamber and incubated at 37°C in a CO_2_ incubator for 6 h. Then the images were taken and migrated cells were quantified. **F**, quantification of data in **E**, the symbols of comparison were same as in A. G, western blot of ADAM10 expression in the above-mentioned cells, with β-actin being used as internal control.

To explore the influence of ADAM10 on SMC properties, we generated HASMCs which stably overexpressed ADAM10 or ADAM10 shRNA via retrovirus-mediated gene transfer. HASMCs infected by pLXSN-vector or pSIREN-shRNA vector were generated as controls. Figure **2** shows the impact of increased ADAM10 expression on HASMCs as compared with non-transduced and vector-transduced cells. MTT assay revealed that overexpression of ADAM10 caused a stepwise increase of HASMCs growth in low glucose, high glucose and high glucose medium with addition of AGE-BSA (100 and 200 µg/ml) (Figure **2A**). Similarly, an elevation in BrdU incorporation was observed in ADAM10-overexpressing HASMCs in these medium (Figure **2B**). In contrast, ADAM10 knockdown resulted in a reduction in HASMC growth and proliferation in both the MTT and BrdU assays. 

Consistent results on migration of the HASMCs were revealed in wound healing and Boyden chamber assays. In comparison with non-transduced and vector-transduced cells, overexpression of ADAM10 induced a significant stepwise increase in migration in both wound healing and Boyden chamber assays from low glucose, high glucose, to high glucose medium with addition of AGE-BSA (Figure **2C, D, E, F**). Conversely, knockdown of ADAM10 significantly decreased migration (Figure **2C, D, E, F**). These data indicated that ADAM10 is implicated in SMCs proliferation and migration under diabetic and non-diabetic conditions.

 To study autocrine role of ADAM10, we treat HASMCs with CM from ADAM10 overexpressing cells. These HASMCs displayed obvious enhancement of metabolic and migratory activity as compared with those treated by CM from vector-transduced cells, however, this enhanced effect was attenuated after addition of anti-ADAM10 antibody (Figure **S3**). 

### Increment of Notch1, 3 and Notch1IC, 3IC detected in ISR than in non-ISR segments in diabetic minipigs

**Figure 3:**
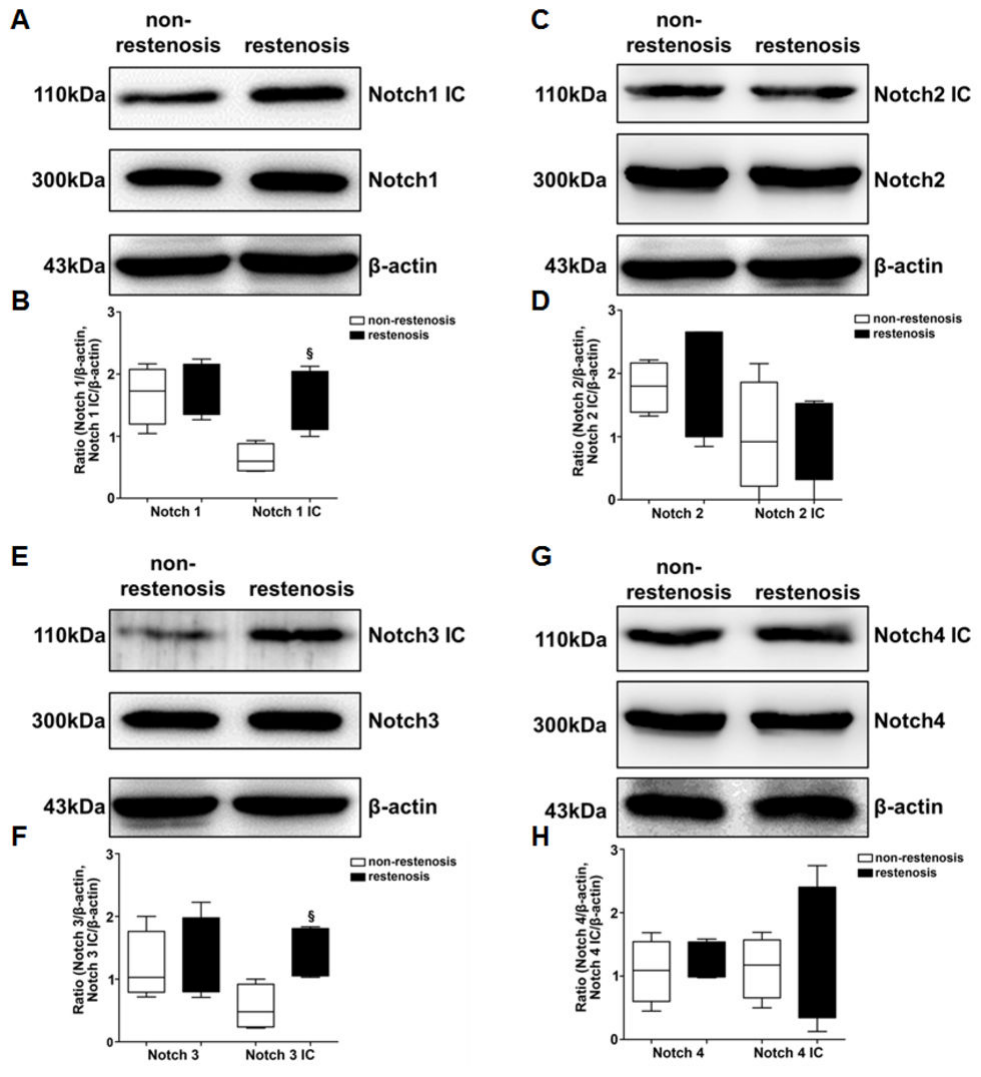
Notch 1 IC and Notch 3 IC levels were significantly increased in ISR versus non-ISR intima of diabetic minipigs. **A**, **B**, **C**, **D**, the protein levels of Notch 1, Notch2, Notch3 and Notch4, and their IC domains were compared by Western blot assay between ISR and non-ISR intima, with quantification (ISR samples, n=6; non-ISR samples, n=24; for each sample, 3 replicates were performed). §P<0.05, vs. non-ISR intima.

Notch pathway modulates cell fate and vascular remodeling and maintains vascular homeostasis. After cleavage by ADAM10 and γ-secretase, intracellular part (IC) of Notch translocates to the nucleus for transcription activation. Of the 4 Notch receptors, Notch1 and Notch3 are predominant in vascular SMCs. Hence, we tested the protein levels of Notch and Notch IC in the intima of stented segments in diabetic minipigs. The levels of Notch1 IC and Notch3 IC were significantly higher in ISR intima than in non-ISR intima (p<0.05), while levels of Notch1 and Notch3 did not reveal any significant differences. Notch2 IC and Notch4 IC level were increased but not to levels reaching statistical significance (Figure **3A, B, C, D**). 

 In order to know the effect of high glucose/AGEs on Notch expression in vascular smooth muscle cells, we treated HASMCs by low glucose, high glucose and high glucose with AGE-BSA (200μg/ml). High glucose with AGE-BSA significantly induced the expression of Notch1 but not Notch3. This treatment also significantly augmented cell metabolic and migratory activities, which could be mitigated by both Notch1 and Notch 3 siRNA (Figure **S4** and **S5**).

### Overexpression of ADAM10 activating Notch signaling pathway in HASMCs

**Figure 4:**
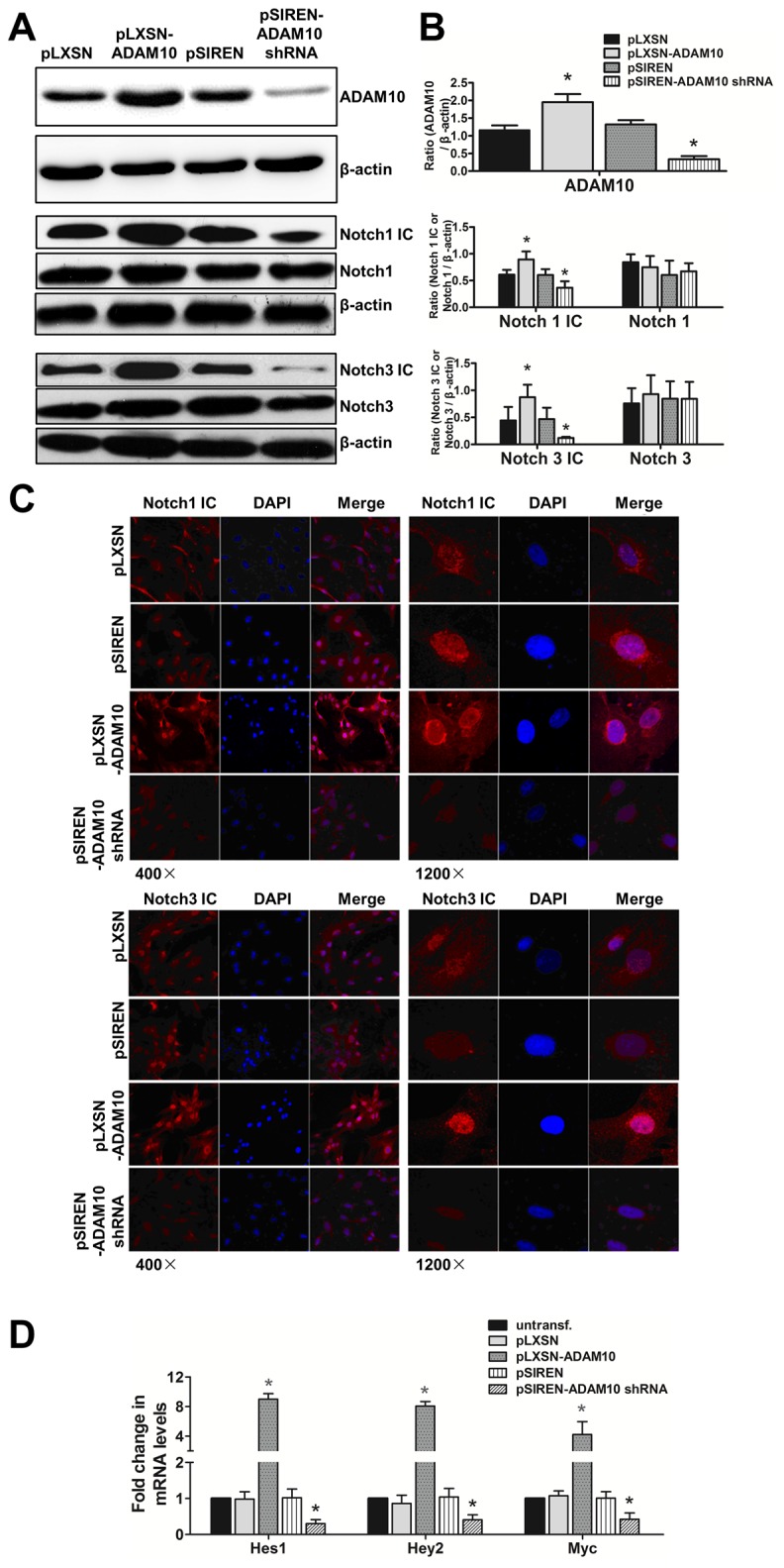
ADAM10 mediates activation of Notch signaling pathway. ADAM10-overexpressing, ADAM10 shRNA-expressing and vector-transduced HASMCs were generated by retrovirus-mediated gene transfer and selection. **A**, the protein levels of ADAM10, Notch 1 IC, Notch 1, Notch 3 IC and Notch 3 were detected in these HASMCs by Western blot, and β-actin was served as internal control. **B**, quantification of the data in **A**, *P<0.05, vs. vector-transduced HASMCs. **C**, cytoplasm and nuclear distribution of Notch 1 IC and Notch 3 IC in these HASMCs were displayed by immuno-fluorescence assay. **D**, the mRNA levels of Notch downstream genes, HES1, HEY2 and myc, were detected by real-time PCR assay, *P<0.05, vs. vector-transduced HASMCs.

To assess the influence of ADAM10 overexpression on Notch activity and to verify Notch homologues involved in ADAM10-mediated activation, we examined endogenous Notch and Notch IC levels of Notch homologues in ADAM10-overexpressing, ADAM10 shRNA-overexpressing and vector-transduced HASMCs. Western blot revealed increased Notch1 IC and Notch3 IC levels upon ADAM10 overexpression (both P<0.05). In contrast, knockdown of ADAM10 significantly suppressed Notch1 IC and Notch3 IC levels (both P<0.05) (Figure **4A, B**). Notch1 and Notch3 levels did not change obviously in the cells. Next, fluorescence immunohistochemistry was performed to detect Notch IC distribution in the cytoplasm and nuclei of these HASMCs. As shown in Figure **4C**, overexpression of ADAM10 elicited stronger fluorescent signals of Notch1 IC and Notch3 IC in the nucleus, reflecting increased nuclear translocation of these molecules, whereas Notch1 IC and Notch3 IC signals were evenly distributed in the vector-transduced cells. In contrast, significantly weaker fluorescent signals of Notch1 IC and Notch3 IC were observed in nuclei and cytoplasm in ADAM10 knockdown HASMCs. 

We studied the mRNA transcription of the target genes of the Notch signaling pathway in these HASMCs (Figure **4D**). Compared with controls, overexpression of ADAM10 induced an increase of HES1, HEY2 and Myc mRNA levels by about 9.0, 8.1 and 4.2 fold, respectively (all P<0.05). In contrast, knockdown of ADAM10 suppressed these genes by about 3.3, 2.4 and 2.4-fold in HASMCs, respectively (all P<0.05). 

### Promotion of ADAM10 overexpression on cell proliferation and migration in part via Notch1 and Notch3 signaling pathway

**Figure 5:**
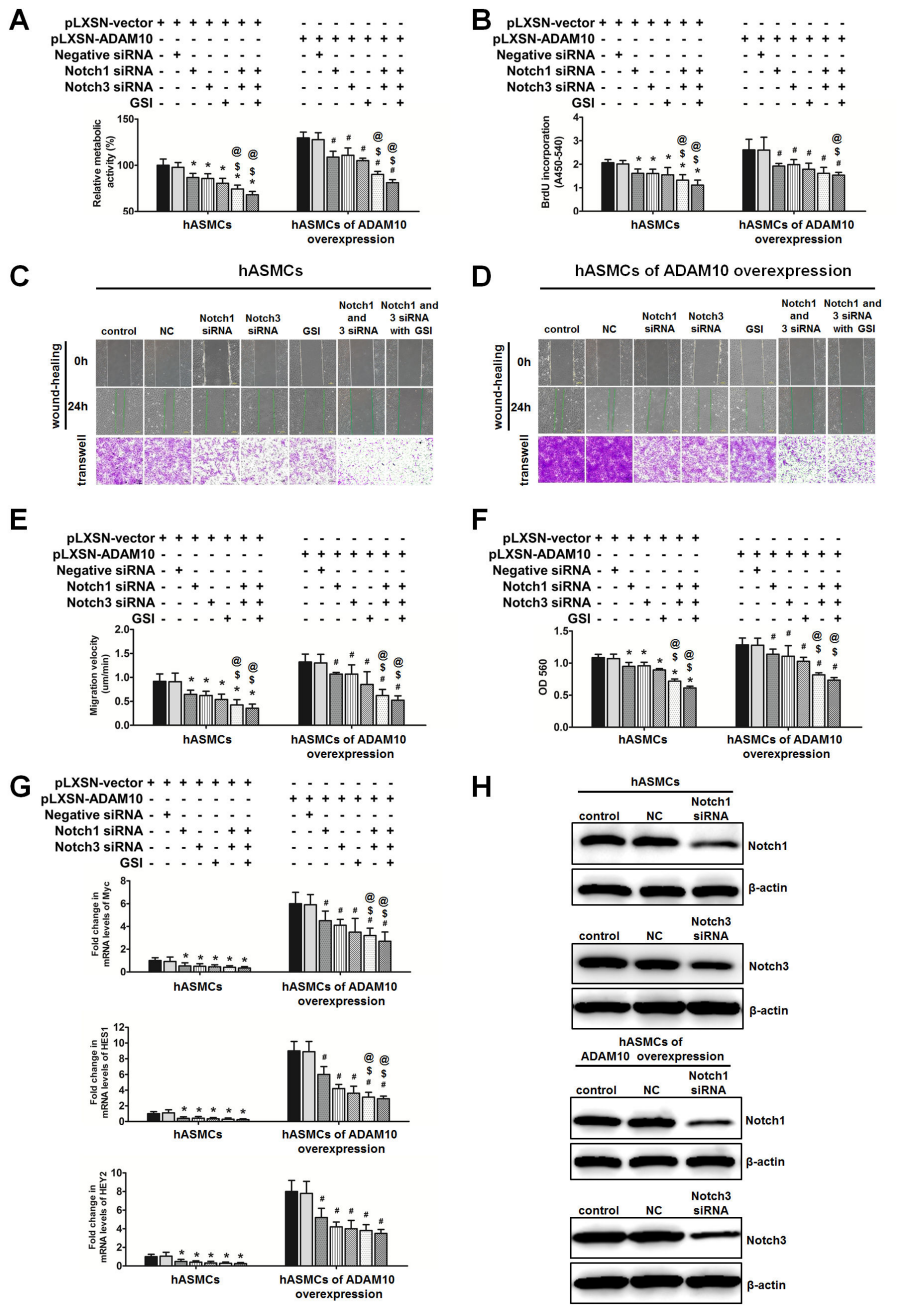
ADAM10 induced proliferation and migration of HASMCs in part through activation of Notch1 and Notch3. **A**, MTT assay were done to measure proliferation ability in HASMCs of pLXSN-vector and pLXSN-ADAM10 overexpression with transfection of control, Notch1 and Notch3 siRNA, in the presence or absence of γ-secretase inhibitor treatment (10 uM). *P<0.05, vs. vector-transduced HASMCs; #P<0.05, vs. ADAM10-overexpressing HASMCs; $P<0.05, vs. HASMCs of vector- and ADAM10-overexpression with Notch1 siRNA transfection; @ P<0.05, vs. HASMCs of vector- and ADAM10-overexpression with Notch3 siRNA transfection. **B**, BrdU proliferation assay was performed in the HASMCs as in A. The symbols of comparison were same as in A. C and D, wound healing and transwell assay were performed in the HASMCs as in A. In wound healing assay, the images were taken before and 24 h after scratch. In transwell assay, the cells were added to the upper chamber, and the chamber was incubated at 37°C in a CO_2_ incubator for 8 h. Then, the migrated cells were stained and the image was taken, followed by quantification by OD 560 nm measurement. **E** and **F**, quantification of wound healing and transwell assay results, respectively. The symbols of comparison were same as in A. G, real-time-PCR analysis was done to examine the mRNA levels of Notch downstream genes in these cells. The symbols of comparison were same as in A. **H**, the protein levels of Notch1, and Notch3 by Notch1 and Notch 3 siRNA transfection versus mock siRNA.

To investigate whether the effects of ADAM10 in promoting proliferation and migration of HASMCs were in part through the Notch pathway, we silenced Notch1 and Notch3 gene in HASMCs, and in ADAM10-overexpressing HASMCs, using siRNA transfection. The results of MTT and BrdU assays demonstrated that Notch1 and Notch3 siRNA significantly decreased cell growth compared to mock siRNA, with combination of Notch1 and Notch3 siRNA causing further decrement than Notch1 and Notch3 siRNA alone (both P<0.05). Combined treatment of γ-secretase inhibitor (10 µM), Notch1 and Notch3 siRNA for 24h produced a more significant decline in proliferation than Notch1 and Notch3 siRNA alone (both P<0.05), and this decreasing was even greater than that of Notch1 and Notch3 siRNA combination although this difference did not reach statistical significance (Figure **5A, B**).

The results of wound healing and Boyden chamber assays were consistent with proliferation data. Both Notch1 and Notch3 knockdown significantly attenuated migration of HASMCs and ADAM10-overexpressing HASMCs as compared with mock siRNA, and combination of Notch1 and Notch3 created more significant mitigation on migration than Notch1 and Notch3 alone (for all comparisons, P<0.05) (Figure **5C, D, E, F**). The presence of γ-secretase inhibitor, Notch1 and Notch3 siRNA together achieved a most remarkable suppression on migration capacity of these HASMCs. Consistent with functional changes, Notch1 and Notch3 knockdown significantly decreased mRNA transcripts of HES1, HEY2 and Myc in HASMCs and in ADAM10-overexpressing HASMCs as compared with mock siRNA. Furthermore, treatment of the HASMCs with combination of the γ-secretase inhibitor, Notch1 and Notch3 siRNA produced a most significant reduction in mRNA levels of these target genes of the Notch signaling pathway (Figure **5G, H**). Taken together, these results indicate that the Notch pathway, in part via Notch1 and Notch3, mediated ADAM10 overexpression-induced proliferation and migration of HASMCs. 

## Discussion

The efficacy of drug-eluting stents in diabetic patients is inferior to that in non-diabetic patients. One major reason is due to high ISR incidence in diabetic patients. We have compared the protein profiles of ISR versus non-ISR segments in the diabetic minipig and uncovered more than 160 proteins with significantly increased levels in ISR samples, including ADAM10. Moreover, the extent of ADAM10 elevation in the ISR is significantly higher in diabetic than in the non-diabetic animals. In cell experiments, ADAM10 overexpression promotes growth and migration of HASMCs through activation of the Notch pathway (in part by Notch1 and Notch3). Although ADAM10 appears to contribute to SMC properties in the non-diabetic state, it is further elevated in diabetic conditions via at least RAGE pathway, which suggests that ADAM10 mechanism may contribute to the enhanced neointimal formation that occurs in diabetes.

Among the identified proteins with significantly increased levels in ISR tissue, AFABP has been found by our group to promote proliferation and migration in HASMCs [3]. Heart-type FABP exerts somehow similar effects on HASMCs as AFABP (data not shown). Other proteins, including bone morphogenetic protein 1, cyclophilin A, high mobility group protein B1, macrophage migration inhibitory factor, S100A11, Thrombospondin-1, TNF receptor-associated factor 6, toll-like receptor 4, and 14-3-3 protein gamma, are involved in promotion of inflammation, ROS production, cell proliferation, cardiovascular remodeling, neurodegeneration and tumor growth in vitro and in vivo through various mechanisms as reported previously (Table **S1**). Vinculin and vitronectin are related to cytoskeletal, extracellular and tissue remodeling. Aldose reductase plays a key role in diabetic metabolism which contributes to diabetic vascular complications. Apolipoprotein B induces vascular inflammation in diabetic and non-diabetic condition. Besides, the relevance of our model system was supported by that in the CM of ISR tissue, we identified heat shock proteins 70 and 27; both proteins were previously shown to promote neointimal hyperplasia after injury [20,21]. On the other hand, we also found several proteins with significantly decreased levels in ISR tissue. These proteins contribute to or are involved in biology of anti-oxidation, anti-inflammation and regulation of cell cycle and cytoskeleton (Table **S2**). 

In our study, ISR tissue culture protocol involves cutting of tissue explants into small pieces followed by several washing steps to remove serum and intracellular proteins before culturing. Because of the cutting, damaged cells slowly lose their contents into medium. Moreover, serum proteins still present in the tissue pieces may diffuse out during culture. Thus, proteomic approach identifies many intracellular proteins, peptides from cleaved fragments of membrane proteins, and plasma proteins in CM, which phenomenon is noted by other studies [22,23]. In addition, it is also possible that small part of the identified ADAM10 molecules are soluble forms generated through ectodomain shedding [24].

 ADAM10 belongs to the transmembrane metalloproteinase family implicated in shedding of dozens of substrates that drive inflammatory pathophysiology; this includes Notch, E-cadherin, EGF, and inflammatory cytokines [6]. The present study has demonstrated the association of ADAM10 overexpression with enhanced growth and migration mediated by the Notch signaling pathway, and the effects of ADAM10 gain of function on metabolic and migratory properties of HASMCs are independent of high glucose and AGEs, suggesting that the ADAM10-Notch mechanism might play a role in ISR formation as in other diseases. Moreover, previous study suggests that chemokine could stimulate ADAM10 mediated effects [25], which contributes to proliferation and pro-inflammatory responses of SMCs. In our study, the finding of high ADAM10 levels in ISR segments in diabetic minipigs is supported by the experiment that high glucose and AGEs induce significantly increased ADAM10 expression in HASMCs. A most recent study has also consistently shown that high glucose level activates another member, ADAM17 [26]. Jointly, we believe that many AGEs, proinflammatory cytokines and upstream signals through RAGE/non-RAGE mediation may trigger the expression of ADAM10, reading to activation of Notch pathways in vascular smooth muscle cells, which results in maintenance of pathophysiological mechanisms of ISR.

 The Notch family has 4 homologues, among which Notch1 and Notch3 are predominantly in vascular SMCs. This study has demonstrated that ADAM10-induced effects are mediated in part via Notch1 and Notch3 in these cells. However, whether Notch2 and Notch4 are related to distinct ADAM10-induced effects in SMCs requires further investigation.

Of note, our data showing modulation of Notch1, Notch3, and Hey2 expression are consistent with several studies that have shown regulation of these molecules in a temporal manner after experimentally induced vascular injury [27-30]. Specifically, Notch-3 and Hey1 have been shown to be upregulated 7 to 14 days after injury compared with uninjured vessels [29]. Furthermore, primary vascular SMC from mice deficient in Hey2 revealed that these cells proliferated at a reduced rate compared with wild-type cells, whereas the overexpression of Hey 1 [30] in SMC led to increased SMC proliferation. 

 It is noteworthy that our study also highlighted S100A11 as a candidate protein for ISR (Table **S1**). This result is consistent with a recent report revealing that S100A11 mediates hypoxia-induced mitogenic factor-stimulated SMC migration, vesicular exocytosis, and nuclear activation [31]. As S100A11 is reported to be a ligand for the receptor for advanced glycation endproducts (RAGE) [32], the present findings validate the proteomic analysis methodology employed in this study. Previous studies showed key roles for RAGE in vascular injury; administration of soluble RAGE or deficiency of RAGE attenuated endothelial-induced neointimal expansion in a non-diabetic murine model [33]. In a rat model, carotid artery blood injury-induced neointimal expansion was suppressed by soluble RAGE in diabetic and non-diabetic animals [34]. Furthermore, as ADAM10 is reported to mediate cleavage and release of extracellular RAGE from the cell surface, distinct roles for ADAM10 in SMC properties in arterial injury may involve regulation of the extracellular forms of RAGE [35]. 

Collectively, this study has demonstrated an increase of ADAM10 expression in ISR segment tissue of diabetic minipigs. Overexpression of ADAM10 promotes proliferation and migration of HASMCs in part through Notch1 and Notch3 signaling pathways. We conclude that ADAM10 may contribute, at least in part, to the exaggerated neointimal formation observed in diabetes.

 We recognize that there is limitation in our study. The number of restenotic vessels is small due to the low ISR rate in both diabetic and non-diabetic groups, making the differential difficult. Further study is required to verify our results and to clarify the impact of ADAM10 in vivo.

## Supporting Information

Figure S1 **Flowchart of the experiments in animals and in cells.**
(TIF)

Figure S2 Activity of ADAM10 of ISR and non-ISR tissues in diabetic and non-diabetic minipigs were analyzed using a commercially available kit. *P<0.05, vs. non-restenosis; $P<0.05, vs. non-diabetic.
(TIF)

Figure S3 **Conditioned medium of ADAM10 overexpression affects proliferation and migration in HASMCs.** HASMCs have been cultured with control or CM from ADAM10 overexpressing cells. MTT and BrdU were used to measure proliferation and migration was detected by wound healing and Transwell assay in HASMCs. *P<0.05, vs. control medium; #P<0.05, vs. conditioned medium of ADAM10 overexpression.
(TIF)

Figure S4 **The effects of high glucose with and without AGE-BSA (200 μg/mL) on expression of Notch1 and 3.** HASMCs were treated by low glucose, high glucose and high glucose with AGE-BSA (200 μg/ml). After 24 hours, cells were collected and Western blot was performed to examine the expression of Notch1 and 3, with β-actin as an internal control. *P<0.05, vs. low glucose treated.
(TIF)

Figure S5 **Notch1 and 3 siRNA attenuated the proliferation and migration of HASMCs induced by high glucose or AGE-BSA.** A, HASMCs were transfected with Notch1 siRNA or control siRNA, and stimulated with high glucose or AGE-BSA (200ug/mL). B, HASMCs were transfected with Notch3 siRNA or control siRNA, and stimulated with high glucose or AGE-BSA (200ug/mL). MTT and BrdU were performed to measure proliferation. Migration activity was detected by wound healing and Transwell assay.*P<0.05, vs. low glucose with NC-transduced; @P<0.05, vs. NC-transduced HASMCs stimulated with high glucose; #P<0.05, vs. NC-transduced HASMCs stimulated with AGE-BSA (200ug/mL).
(TIF)

Table S1 **Major proteins with increased levels in diabetic ISR segments and with biological functions potentially related to ISR pathophysiology.**
(DOC)

Table S2 **Major proteins with decreased levels in diabetic ISR segments and with biological functions potentially related to ISR pathophysiology.**
(DOC)

Table S3 **The primers and shRNA sequence information for plasmid construction.**
(DOC)

Table S4 **The primers used in real-time PCR.**
(DOC)

Table S5 **Angiographic and IVUS assessment at 6 months. Abreviation: MLD, minimal lumen diameter; #P<0.05 vs. non-diabetic group, ##P<0.01 vs. non-diabetic group.**
(DOC)
