# Supplementary figures and images for: Increase of ADAM10 Level in Coronary Artery In-Stent Restenosis Segments in Diabetic Minipigs: High ADAM10 Expression Promoting Growth and Migration in Human Vascular Smooth Muscle Cells via Notch 1 and 3
Source: PLoS One. 2013 Dec 27;8(12):e83853. doi: 10.1371/journal.pone.0083853 (PMC3873985; doi:10.1371/journal.pone.0083853)

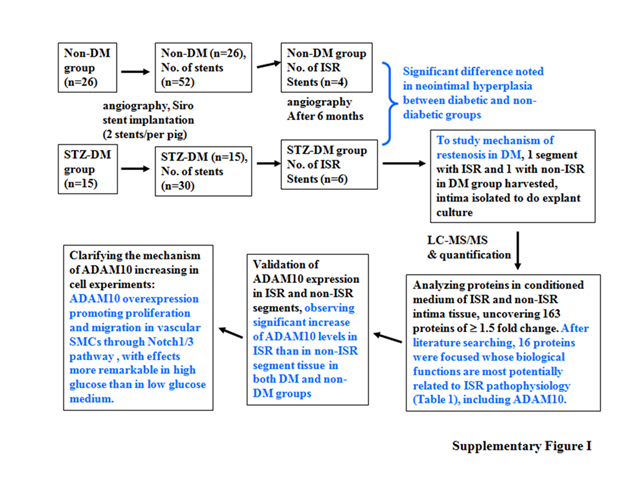

Supplement: Figure S1 — Flowchart of the experiments in animals and in cells. (TIF) [file pone.0083853.s001.tif]

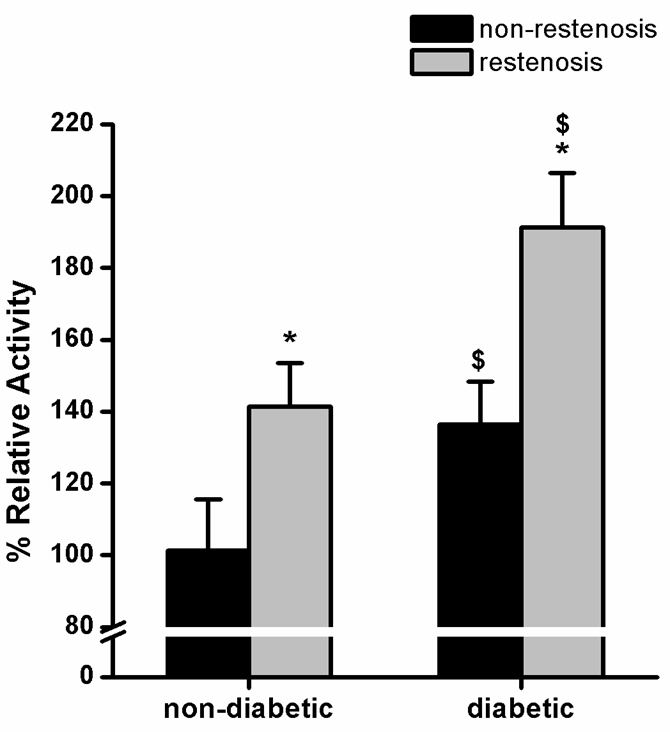

Supplement: Figure S2 — Activity of ADAM10 of ISR and non-ISR tissues in diabetic and non-diabetic minipigs were analyzed using a commercially available kit. *P<0.05, vs. non-restenosis; $P<0.05, vs. non-diabetic. (TIF) [file pone.0083853.s002.tif]

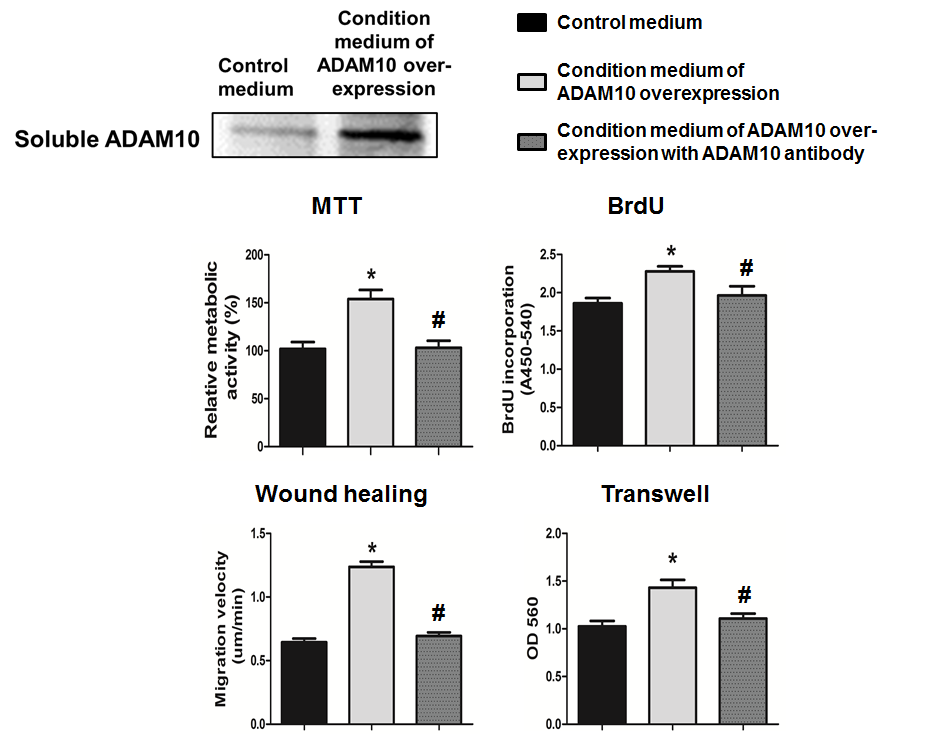

Supplement: Figure S3 — Conditioned medium of ADAM10 overexpression affects proliferation and migration in HASMCs. HASMCs have been cultured with control or CM from ADAM10 overexpressing cells. MTT and BrdU were used to measure proliferation and migration was detected by wound healing and Transwell assay in HASMCs. *P<0.05, vs. control medium; #P<0.05, vs. conditioned medium of ADAM10 overexpression. (TIF) [file pone.0083853.s003.tif]

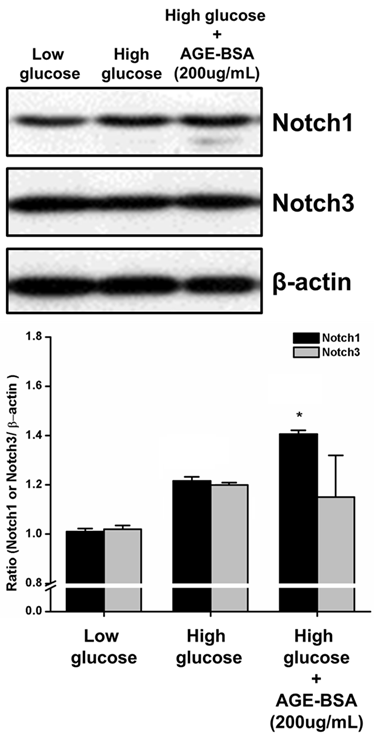

Supplement: Figure S4 — The effects of high glucose with and without AGE-BSA (200 μg/mL) on expression of Notch1 and 3. HASMCs were treated by low glucose, high glucose and high glucose with AGE-BSA (200 μg/ml). After 24 hours, cells were collected and Western blot was performed to examine the expression of Notch1 and 3, with β-actin as an internal control. *P<0.05, vs. low glucose treated. (TIF) [file pone.0083853.s004.tif]

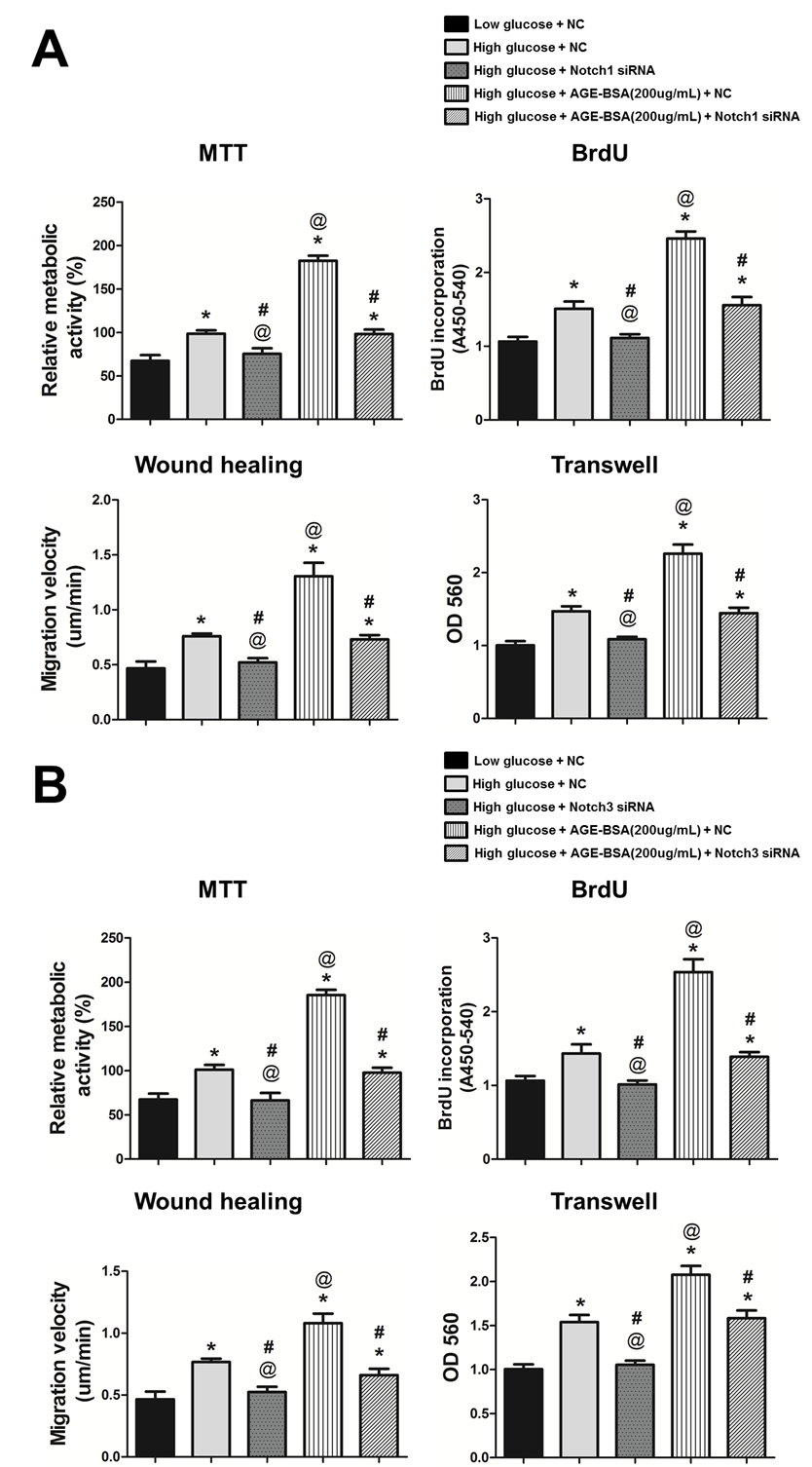

Supplement: Figure S5 — Notch1 and 3 siRNA attenuated the proliferation and migration of HASMCs induced by high glucose or AGE-BSA. A, HASMCs were transfected with Notch1 siRNA or control siRNA, and stimulated with high glucose or AGE-BSA (200ug/mL). B, HASMCs were transfected with Notch3 siRNA or control siRNA, and stimulated with high glucose or AGE-BSA (200ug/mL). MTT and BrdU were performed to measure proliferation. Migration activity was detected by wound healing and Transwell assay.*P<0.05, vs. low glucose with NC-transduced; @P<0.05, vs. NC-transduced HASMCs stimulated with high glucose; #P<0.05, vs. NC-transduced HASMCs stimulated with AGE-BSA (200ug/mL). (TIF) [file pone.0083853.s005.tif]
