# Supplementary material for: Increase of ADAM10 Level in Coronary Artery In-Stent Restenosis Segments in Diabetic Minipigs: High ADAM10 Expression Promoting Growth and Migration in Human Vascular Smooth Muscle Cells via Notch 1 and 3
Source: PLoS One. 2013 Dec 27;8(12):e83853. doi: 10.1371/journal.pone.0083853 (PMC3873985; doi:10.1371/journal.pone.0083853)
Supplement: Table S1 — Major proteins with increased levels in diabetic ISR segments and with biological functions potentially related to ISR pathophysiology. (DOC) [file pone.0083853.s006.doc]

**Table S1. Major proteins with increased levels in diabetic ISR segments and with biological functions potentially related to ISR pathophysiology**

| **Accession**  **Number** | **Protein Name** | **Function** | **References** |
| --- | --- | --- | --- |
| B3VKE8 | ADAM10 | ADAM10-mediated shedding of substrates promoting inflammation,vascular remodeling, neovascularisation, neurodegenerative disorder and tumor growth | Eur J Cell Biol 2012;91:472  Circulation 2009;119:2686  Eur J Cell Biol 2011;90:527 |
| O97675 | Adipocyte fatty acid binding protein | related to inflammation, ROS production, atherosclerosis and metabolic syndrome, and in-stent restenosis | Am J Physiol Heart Circ Physiol 2012;302:H1231  Arterioscler Thromb Vasc Biol 2013;33:572 |
| O02772 | Heart-type fatty acid binding protein | acute myocardial ischemia, and stroke | Transl Res 2012;159:252  Mol Diagn 2005;9:1 |
| P80276 | Aldose reductase | a key player in mediating diabetic complications | Cardiovasc Hematol Agents Med Chem 2012;10:234 |
| F1SCV8 | apolipoprotein B | participating in atherosclerosis | Curr Opin Lipidol 2012;23:422 |
| Q95L23 | bone morphogenetic protein 1 | activating TGF beta superfamily in regulation of extracellular matrix | Matrix Biol 2007;26:508 |
| P62936 | Cyclophilin A | a secreted oxidative-stress-induced immunophilin with diverse functions, inflammation, ROS production, cardiovascular remodeling, aortic aneurysms, and atherosclerosis | Can J Physiol Pharmacol 2012;90:1005  Circulation 2008;227:3088 |
| F2Z594 | High mobility group protein B1 | involved in atherosclerosis, inflammation, ROS production, cardiovascular remodeling, injury and tumor | Autoimmun Rev 2012;11:909  Ann N Y Acad Sci 2011;1243:88 |
| P80928 | Macrophage migration inhibitory factor | involved in inflammation, immune response, and cell growth | Ann N Y Acad Sci 2012;1271:53 |
| P31950 | S100A11 | one of the RAGE ligands, related to cell inflammation and migration | Curr Mol Med 2007;7:711 |
| Q5U903 | Thrombospondin-1 | involved in angiogenesis, cancer and inflammation | Mediators Inflamm 2011;2011:296069 |
| Q70AC9 | TNF receptor-associated factor 6 | involved in innate and adaptive immunity, bone metabolism, development of central nervous system | Adv Exp Med Biol 2007;597:122 |
| Q68Y56 | Toll-like receptor 4 | participating in ischemic reperfusion injury, atherosclerosis, inflammation, ROS production, and innate immunity | Int Rev Immunol 2012;31:379 |
| P26234 | Vinculin | related to actomyosin machinery, control of cell proliferation, and cell-cell junctions | J Cell Biol 2012;196:641  Eur J Cell Biol 2011;90:157 |
| P48819 | Vitronectin | associated with vessel wall and tumor cells upon tissue remodeling, injury/repair, or under disease conditions | Semin Thromb Hemost 2011;37;408 |
| P61983 | 14-3-3 protein gamma | related to cell growth, cytoskeletal regulation, differentiation, tumor progression and neurodegeneration | Semin Cell Dev Biol 2011;22:681  Semin Cell Dev Biol 2011;22:663 |

Identified proteins are listed in alphabetic order.
