# Supplementary material for: Increase of ADAM10 Level in Coronary Artery In-Stent Restenosis Segments in Diabetic Minipigs: High ADAM10 Expression Promoting Growth and Migration in Human Vascular Smooth Muscle Cells via Notch 1 and 3
Source: PLoS One. 2013 Dec 27;8(12):e83853. doi: 10.1371/journal.pone.0083853 (PMC3873985; doi:10.1371/journal.pone.0083853)
Supplement: Table S2 — Major proteins with decreased levels in diabetic ISR segments and with biological functions potentially related to ISR pathophysiology. (DOC) [file pone.0083853.s007.doc]

**Table S2. Major proteins with decreased levels in diabetic ISR segments and with biological functions potentially related to ISR pathophysiology**

| **Accession**  **Number** | **Protein Name** | **Function** | **References** |
| --- | --- | --- | --- |
| P18648 | Apolipoprotein A-I | playing a role in anti-inflammation, anti-oxidation anti-atherosclerosis and lipid transportation | EMBO Mol Med 2012;4:251  Curr Atheroscler Rep 2008;10:171 |
| P20305 | Gelsolin | involved in immunomodulation, and in suppression of tumor metastasis and collagen remodeling | Am J Physiol Cell Physiol 2010;299:C1516  Cancer Res 2013;73:3625  Am J Physiol Cell Physiol 2007;293:C1049 |
| K9IWF9 | IQGAP1 | involved in S1P-mediated vascular barrier protection | Am J Physiol Lung Cell Mol Physiol 2012;303:L12 |
| A4US67 | Paraoxonase 1 | capable of anti-oxidation and inhibiting atherosclerosis and inflammation | Curr Opin Lipidol 2009;20:265  Am J Cardiovasc Drugs 2004;4:211  Adv Exp Med Biol 2010;660:29 |
| F1SDX9 | Peroxiredoxin-2 | a peroxidase, inhibiting atherogenic responses in vascular and inflammatory cells. | Circ Res 2011;109:739  FEBS J 2009;276:2469 |
| Q19QU3 | RAN-binding domain-containing protein 2 (ZRANB2) | suppressor of bone morphogenetic protein signaling | J Cell Biochem 2012;113:808 |
| F1ST01 | Selenium-binding protein 1 | possessing tumor suppression function, being a biomarker for prediction of hepatocellular carcinoma prognosis | PLoS One 2009;4:e7774  Clin Cancer Res 2012;18:3042 |
| A0PA01 | Serine protease inhibitor 9 | having inhibitory effects on endothelial cell apoptosis and anti-inflammatory effects | Circ Res 2013;112:771-780  Inflamm Res 2010;59:679-687 |
| P04178 | Superoxide dismutase [Cu-Zn] | playing a role in redox signaling, an d modulation of vascular function and diseases | Antioxid Redox Signal 2011;15:1583  Free Radic Biol Med 2010;48:1565 |
| A4PES0 | Wee1-line protein kinase 2 (Wee1B kinase) | involved in the regulation of cell cycle | Mol Biol Cell 2005;16:5749  Mol Med Rep 2013;7:1929-1937 |

Identified proteins are listed in alphabetic order.
