# Supplementary material for: Increase of ADAM10 Level in Coronary Artery In-Stent Restenosis Segments in Diabetic Minipigs: High ADAM10 Expression Promoting Growth and Migration in Human Vascular Smooth Muscle Cells via Notch 1 and 3
Source: PLoS One. 2013 Dec 27;8(12):e83853. doi: 10.1371/journal.pone.0083853 (PMC3873985; doi:10.1371/journal.pone.0083853)
Supplement: Table S3 — The primers and shRNA sequence information for plasmid construction. (DOC) [file pone.0083853.s008.doc]

**Table S3. The primers and shRNA sequence information for plasmid construction**

|  | **senquence** |
| --- | --- |
| Clony primers of ADAM10 | Sense: ATCTCGAGATGGTGTTGCTGAGAGTG  Antisense: AAGGATCCTTAGCGTCTCATGTGTCC |
| Target sequence for ADAM10 knockdown | AAGCTGTGATTGCCCAGATAT |
| Target sequence for Notch1 knockdown | AAGTGTCTGAGGCCAGCAAGA |
| Target sequence for Notch2 knockdown | AACATCAACGAGTGCTCCAGC |
| Target sequence for Notch3 knockdown | AACACCTATAACTGCCAGTGC |
| Target sequence for Notch4 knockdown | AACCCTGTGCCAATGGAGGCA |
