# Supplementary material for: Increase of ADAM10 Level in Coronary Artery In-Stent Restenosis Segments in Diabetic Minipigs: High ADAM10 Expression Promoting Growth and Migration in Human Vascular Smooth Muscle Cells via Notch 1 and 3
Source: PLoS One. 2013 Dec 27;8(12):e83853. doi: 10.1371/journal.pone.0083853 (PMC3873985; doi:10.1371/journal.pone.0083853)
Supplement: Table S4 — The primers used in real-time PCR. (DOC) [file pone.0083853.s009.doc]

**Table S4. The primers used in real-time PCR**

| **Gene Name** | **Product size (bp)** | **Sense primer** | **Anti-sense primer** |
| --- | --- | --- | --- |
| β-actin | 176 | CGTTGACATCCGTAAAGACC | TAGAGCCACCAATCCACACA |
| Hes1 | 221 | CTGCATGACCCAGATCAATG | AGCCTCCAAACACCTTAGCC |
| Hey2 | 241 | GATGCTTCAGGCAACAGG | GTGGAGCGGATGATGGTG |
| Myc | 179 | ACACCCTTCTCCCTTCG | CCGCTCCACATACAGTCC |
