# Supplementary material for: Increase of ADAM10 Level in Coronary Artery In-Stent Restenosis Segments in Diabetic Minipigs: High ADAM10 Expression Promoting Growth and Migration in Human Vascular Smooth Muscle Cells via Notch 1 and 3
Source: PLoS One. 2013 Dec 27;8(12):e83853. doi: 10.1371/journal.pone.0083853 (PMC3873985; doi:10.1371/journal.pone.0083853)
Supplement: Table S5 — Angiographic and IVUS assessment at 6 months. Abreviation: MLD, minimal lumen diameter; #P<0.05 vs. non-diabetic group, ##P<0.01 vs. non-diabetic group. (DOC) [file pone.0083853.s010.doc]

**Table S5**. Angiographic and IVUS assessment at 6 months

|  | Diabetic group  (n=15, stent No.=30) | Non-diabetic group  (n=26, stent No.=52) |
| --- | --- | --- |
| Left anterior descending artery | 14 | 20 |
| Left circumflex artery | 3 | 12 |
| Right coronary artery | 13 | 20 |
| Reference vessel diameter (mm) | 2.59 ± 0.32 | 2.70 ± 0.25 |
| Stent deploy pressure (atm) | 13.4 ± 3.0 | 14.4 ± 3.3 |
| Final MLD (mm) | 3.04 ± 0.30 | 3.16 ± 0.22 |
| Stent to vessel ratio | 1.11 ± 0.12 | 1.17 ± 0.12 |
| In-stent MLD (mm) | 2.45 ± 0.32# | 2.85 ± 0.22 |
| In-segment MLD (mm) | 2.43 ± 0.29# | 2.80 ± 0.30 |
| In-stent diameter stenosis (%) | 40.4 ± 24.0# | 20.0 ± 18.1 |
| In-segment diameter stenosis (%) | 43.1 ± 23.9# | 21.5 ± 16.0 |
| In-stent late loss (mm) | 0.33 ± 0.19# | 0.10 ± 0.09 |
| In-segment late loss (mm) | 0.31 ± 0.18# | 0.11 ± 0.15 |
| In-stent restenosis >50%  (number of stent) | 6 | 4 |
| Neointimal volume (mm3) | 21.9 ± 18.7## | 3.90 ± 2.95 |
| %intimal hyperplasia | 26.7 ± 19.2## | 7.4 ± 6.5 |

Abreviation: MLD, minimal lumen diameter; #P<0.05 vs. non-diabetic group, ##P<0.01 vs. non-diabetic group
